# The Relationship among COVID-19 Information Seeking, News Media Use, and Emotional Distress at the Onset of the Pandemic

**DOI:** 10.3390/ijerph182413198

**Published:** 2021-12-14

**Authors:** Juwon Hwang, Porismita Borah, Dhavan Shah, Markus Brauer

**Affiliations:** 1 School of Media and Strategic Communications, Oklahoma State University, Stillwater, OK 74078, USA; 2 Edward R. Murrow College of Communication, Washington State University, Pullman, WA 99163, USA; p.borah@wsu.edu; 3 School of Journalism and Mass Communication, University of Wisconsin-Madison, Madison, WI 53706, USA; dshah@wisc.edu; 4 Department of Psychology, University of Wisconsin-Madison, Madison, WI 53706, USA; markus.brauer@wisc.edu

**Keywords:** information seeking, television news use, emotional distress, COVID-19, social media news use

## Abstract

Although several theories posit that information seeking is related to better psychological health, this logic may not apply to a pandemic like COVID-19. Given uncertainty inherent to the novel virus, we expect that information seeking about COVID-19 will be positively associated with emotional distress. Additionally, we consider the type of news media from which individuals receive information—television, newspapers, and social media—when examining relationships with emotional distress. Using a U.S. national survey, we examine: (1) the link between information seeking about COVID-19 and emotional distress, (2) the relationship between reliance on television, newspapers, and social media as sources for news and emotional distress, and (3) the interaction between information seeking and use of these news media sources on emotional distress. Our findings show that seeking information about COVID-19 was significantly related to emotional distress. Moreover, even after accounting for COVID-19 information seeking, consuming news via television and social media was tied to increased distress, whereas consuming newspapers was not significantly related to greater distress. Emotional distress was most pronounced among individuals high in information seeking and television news use, whereas the association between information seeking and emotional distress was not moderated by newspapers or social media news use.

## 1. Introduction

The COVID-19 pandemic has not only disrupted basic everyday activities, but also fostered emotional distress [1,2,3]. After isolated cases and clusters started appearing in the early months of 2020, by March the U.S. saw rapidly increasing case counts indicating community transmission [4]. With COVID-19 declared a pandemic by the World Health Organization on 11 March and a national emergency by the Trump administration on 13 March, states implemented shelter-in-place or stay at home orders [5], potentially contributing to unease and mental distress. Research documenting the extent of emotional distress during the COVID-19 pandemic is rapidly emerging (e.g., [1,2,6,7]). This research builds on work showing that there is a significant relationship between the occurrence of infectious disease outbreaks and negative psychological consequences. For example, people are likely to develop greater incidence of depression [8], psychological distress [8,9], and anxiety [10] during pandemics.

Since the COVID-19 outbreak, individuals have sought to understand basic information related to the virus such as its impact, effective treatment, and vaccine development [11]. The lack of predictability, the rising number of confirmed cases and deaths, and changing health guidelines led wide swaths of the public to seek information about the pandemic [12]. In fact, according to a report from the Pew Research Center, 70% of U.S. citizens searched online for information about the coronavirus in the early months of the pandemic [13].

Several theories and empirical findings suggest a positive relationship between information seeking and emotional distress especially during crises. In fact, information seeking about negative events such as natural disasters [14,15], terrorism [16,17], and pandemics [18] is linked to emotional distress. Moreover, following the reliance on heuristics under uncertainty [19,20], an unprecedented amount of information may cause emotional distress. So, when confronted by intense media coverage about COVID-19, people may perceive higher levels of threat, which, in turn, may trigger higher stress. Finally, people might be incapable of avoiding information seeking because of the need-to-know basic information, such as the symptoms of infection.

Information seeking, as a proxy for attention paid to COVID-19 news, may interact with the news source through which information is consumed. Specific combinations of attention and exposure may also be related to emotional distress, with certain types of news sources more likely to spur strong emotions (e.g., [21,22]). Particularly for television, attention must be considered alongside exposure [23,24], especially considering the unique capabilities of video for conveying emotions [17]. This is because news on television features vivid images, motion and sound, whereas newspapers emphasize text and limited use of visuals. Taking into account the medium through which people find news during the COVID-19 pandemic may explain distress mechanisms. Furthermore, the types of media through which individuals find news may moderate the relationship between information seeking and emotional distress. For example, if an individual tends to rely on television as a source for news and is seeking information about COVID-19, the modality of this medium may amplify the association between information seeking and emotional distress beyond the direct relationship of each factor.

Using a U.S. national survey, we examine: (1) the link between information seeking concerning the COVID-19 pandemic and individuals’ emotional distress, (2) the relationship between reliance on television, newspapers, and social media as sources for news exposure on emotional distress during the pandemic after accounting for COVID-19 information seeking, and (3) the interaction between information seeking about COVID-19 and use of these news media sources on emotional distress. In doing so, our study attempts to understand the psychological toll of information seeking and news media use during an ongoing pandemic. Understanding these relationships is critical because seeking information via news media has been especially important during the COVID-19 pandemic. However, at the same time, the contentiousness of partisan news and the presentational styles of some media forms about the pandemic could lead to emotional distress. In this study, we attempt to unpack these relationships.

### 1.1. Information Seeking and Emotional Distress

Information seeking is the process by which individuals “purposefully make an effort to change their state of knowledge” ([25], p. 549; [26]). Both individuals’ motivation to seek information and media coverage on the specific topic tend to increase during crises [11,27]. Due to the novel nature of COVID-19 especially, information about COVID-19 has been placed at the forefront of much of the media [28]. The pandemic dominated news content during the first half of 2020 [27,28]. Given its prevalence and potential impact, theories and studies suggest a positive relationship between information seeking and emotional distress during a major pandemic like the one caused by COVID-19.

First, information seeking about certain events using media might be related to negative emotions [14,15,16,18]. This is particularly evident in studies on information seeking about traumatic events, such as disasters [14,15], terrorism [16,17], and pandemics [18]. When a traumatic event occurs, individuals often attempt to reduce uncertainty about the event by engaging in information seeking. However, efforts to learn more about the traumatic event may be linked with negative emotional reactions to said event [16]. In the case of September 11, people sought to alleviate uncertainty by seeking information about the event, and this behavior was related to a variety of negative emotions [16], due partially to underlying uncertainty about the event [16] and the ways in which media covered it. This same logic can be applied to the global COVID-19 pandemic, as the uncertainty and unpredictability of COVID-19 poses risks to individuals’ mental health (e.g., [1,2,6,7]). 

Second, the reliance on heuristics under uncertainty [19,20] also helps explain why individuals are stressed with COVID-19 information seeking. Uncertain people tend to refer to heuristics, or mental shortcuts. According to the availability heuristic [19,20], there are situations in which people assess the likelihood of an event by how readily examples come to mind [20]. People may perceive higher levels of threat when the events are salient and memorable, with vivid evidence [20]. Media coverage is one way to make the event available in people’s minds, ensuring that people are easily able to retrieve information concerning that event. In the case of COVID-19, there has been a remarkable amount of media coverage, making it available to most people who seek information about the pandemic. This higher availability of information about the global pandemic may cause higher levels of stress.

Finally, under certain circumstances, individuals might choose to avoid information seeking when they perceive that more knowledge might lead to distress [29,30,31]. However, avoiding information seeking might not always be an option. In the case of the COVID-19 pandemic, an already unprecedented amount of uncertainty has been increased by the spread of conspiracy theories and misinformation [12]. Even if people know consuming information leads to stress, they might not have a choice to avoid it, due to the need to find basic answers like safe ways to get groceries or symptoms of COVID-19 infection. The evolving nature of the pandemic meant critical information frequently changed, requiring active information seeking to keep up with changing facts and guidelines, despite the potential distress.

Since the onset of the COVID-19 pandemic, there has been a growing body of the literature dealing with information seeking and emotional distress (e.g., [32,33,34]). The previous findings, however, are somewhat inconsistent. While some studies showed that information seeking is significantly related to anxiety [33] or information overload [34], other studies indicated that high levels of information seeking are associated with higher levels of well-being and risk perception [32]. To address the inconsistency in the literature, we examine the relationship between COVID-19 information seeking and emotional distress using a large U.S. national sample. Despite the mixed findings, based on the aforementioned discussion, we propose our first hypothesis as follows:

**Hypothesis** **1.** *A higher level of COVID-19 information seeking is positively related to emotional distress during the COVID-19 pandemic.*

### 1.2. Information Seeking, General News Media Use, and Emotional Distress

The association between news media use and individuals’ emotional distress concerning COVID-19 may depend on the modality of the news medium from which individuals get information. This idea is associated with Marshall McLuhan’s [35] early work, which emphasizes the differences in media modalities. Studies in the McLuhan tradition focus on “the differences in the physical modalities of video versus print and offer evidence to show that video is the most effective medium for communicating information” ([36], p. 79). Indeed, audiovisual media such as television have been found to have a greater impact on information recall and counterarguing compared to print media [37,38]. Audiovisual media attract attention and stimulate involvement [39]. By contrast, the presentation of information in print modalities seems to reduce the ability to foster emotional arousal [17]. In line with this research, we consider how consuming news via television, newspapers, and social media may be related to emotional distress beyond information seeking concerning COVID-19. Furthermore, the link between COVID-19 information seeking and emotional distress may not be the same for all news consumers. Instead, the type of media through which individuals find general news may interact with information seeking about COVID-19 to explain emotional distress.

### 1.3. Television News

Because television news, as an audiovisual medium, may require fewer cognitive skills than print media, it is more likely to capture the attention of people who possess fewer cognitive skills [36]. Its combination of audio and visual tracks, repeated usage of strong imagery, and news anchors’ visible displays of emotion may elicit emotional responses in news viewers [40,41]. Indeed, television news is more emotionally arousing than newspaper stories [17]. Previous studies show the strong association between television news consumption and viewers’ negative emotional outcomes (e.g., [22,42,43,44,45,46]). However, this association may be due to the kind of thinking television viewers have to do to make sense of a cultural experience [47]. An experimental study showed that exposure to a random newscast triggered increased negative emotions, and manifested in heightened anxiety, total mood disturbance, and decreased positive affect [45]. The emotional distress may be more intense after exposure to televised reports of exceptionally negative events [46]. In addition, a systematic review of literature on disaster news viewing and psychological outcomes linked consumption of televised news with a range of negative emotions [22]. Specifically, television viewing in the context of terrorism was associated with posttraumatic stress (PTS; [43]), stress reactions [44], and negative emotional responses [17]. Given that the technical features of television are particularly appropriate for evoking emotional responses, we propose the following hypothesis:

**Hypothesis** **2.** *Accounting for information seeking about COVID-19, consuming news via television will be related to increased emotional distress*.

**Hypothesis** **3.** *The association between COVID-19 information seeking and emotional distress will be moderated by television news use, with the association between information seeking and emotional distress stronger for individuals with higher television news use.*

### 1.4. Newspapers

In contrast to television news, newspapers and other print media’s lack of visual, motion, and audio cues reduce a reader’s sense of presence. Moreover, newspapers and newsmagazines provide in-depth, thematic, and analytic coverage on issues and matters of public interest, with less emotion-laden language compared to television news, which tends to combine an emphasis on emotional content with episodic coverage [17]. These characteristics position newspapers as a less emotionally arousing medium.

Research shows that newspapers evoke weaker emotions in readers when compared with the effect of television news on viewers (e.g., [36]). For example, while people who watched television news experienced stronger emotions related to terrorist attacks, newspaper usage was not a significant factor in explaining individuals’ emotional responses [17]. Similarly, according to a systematic review of literature on various forms of disaster media and psychological outcomes [22], none of the reviewed studies showed significant associations between newspaper use and psychological outcomes such as depression, stress, and anxiety. Given that newspaper stories feature fewer emotion-laden visuals, we propose the following hypothesis:

**Hypothesis** **4.** *Accounting for information seeking about COVID-19, consuming news via newspapers will be related to decreased emotional distress.*

**Hypothesis** **5.** *The association between COVID-19 information seeking and emotional distress will be moderated by newspaper use, with the association between information seeking and emotional distress weaker for individuals with higher newspaper use.*

### 1.5. Social Media News

Finally, with the rise of mobile technology, accessing news and information on social media has become commonplace and frequent [48]. In 2019, 53% of U.S. adults received news from social media, up from 47% in 2018 [48]. While social media share traditional media’s ability to provide news to users [49], social media have unique characteristics that are markedly different from traditional forms of media. First, while traditional media are defined as either textual media (e.g., newspapers) or audiovisual media (e.g., television news), social media provide a combination of modality (i.e., both textual and audiovisual mode). Social media users can share dramatic multimedia clips about apparent health risks using video sharing sites such as YouTube [21], many of which are unverified. Second, social media are highly personalized platforms, connecting users with similar interests, often with personal or professional relationships [50]. Social media can reflect a social endorsement from ‘people like me’ via established social contacts (e.g., Facebook) or through like-minded individuals (e.g., Twitter). This aspect of social media allows for the rapid spread of misinformation [51] because users rely on social endorsement [52] rather than verified information. According to a report from the Pew Research Center, those who get most of their news from social media reported seeing at least some misinformation about the COVID-19 outbreak [53]. These same news consumers said media have exaggerated the threat posed by COVID-19.

All of these features of social media may have caused the discourse on social media concerning COVID-19 to be emotionally arousing and stressful. Prior research shows higher levels of emotional distress among social media news users than other media users. One study showed that individuals who consumed news solely from news feeds, or news feeds plus online news websites, had higher rates of neuroticism (feeling anxious or depressed/worried) compared to participants consuming news exclusively offline [54]. Another study compared post-traumatic stress one month after Hurricane Sandy among those who learned about the disaster through traditional media (television, newspapers, and radio) versus those who learned about it through social media (Facebook, YouTube, and Twitter; [21]). The researchers found that posttraumatic stress was higher in those using social media relative to those using only traditional media. This could be because social media exert direct and personal impact, owing to the type of content being shared, compared to traditional media that provide more ‘objective’ information.

The modality of social media (i.e., combination of audiovisual and textual information), its endorsement functions (i.e., likes, shares), and the lack of gatekeeping of information sources circulated on social media may strengthen emotional responses in those who rely on this as a source for news. Accordingly, we predict the following hypotheses:

**Hypothesis** **6.** *Accounting for information seeking about COVID-19, consuming news via social media will be related to increased emotional distress.*

**Hypothesis** **7.** *The association between COVID-19 information seeking and emotional distress will be moderated by social media news use, with the association between information seeking and emotional distress stronger for individuals with higher social media news use.*

## 2. Methods

### 2.1. Data

Responding to widespread “community transmission” within the U.S. (the virus being transmitted by individuals with no travel history) in mid-March 2020, a survey was rapidly assembled and collected by a cross-disciplinary team of researchers at a large Midwestern university. Data were collected from 26 March to 1 April 2020 using a Qualtrics panel, a representative sample of U.S. residents based on a pre-recruited pool of panelists (*n* = 2251). This sample also contained a probability sub-sample of residents of the Midwestern state in which the sponsoring university is located. Participants had a mean age of 46.6 (*SD* = 17.0), 58.2% were female, and 68.9% were white. In terms of education, 22.4% had some high school education or a high school diploma, 21.4% had some college education but no degree, 35.8% had an associate’s or bachelor’s degree, and 20.4% had an advanced degree.

### 2.2. Measures

Emotional distress. Participants indicated the extent to which they experienced the following feelings since they became aware of the COVID-19 outbreak: (1) “Overwhelmed,” (2) “Anxious,” and (3) “Afraid about what might happen.” Responses options ranged on a 5-point scale from 1 = *not at all* to 5 = *very much* (*M* = 3.45, *SD* = 1.08, Cronbach’s α = 0.83).

COVID-19 information seeking. Participants were asked to answer a single-item about how frequently they had sought news updates about COVID-19 on a 5-point scale from 1 = *never* to 5 = *any time I have the chance* (*M* = 3.77, *SD* = 1.12).

General news media usage. General news media usage separated by media type, was assessed by the question “How often do you get news from the following sources?” rated on a 5-point scale from 1 = *never* to 5 = *every day*. Television news media usage was measured with the item, “National network news, such as ABC, NBC, CBS” (*M* = 3.62, *SD* = 1.38). Newspaper news media usage was measured with the item, “newspaper and news magazines” (*M* = 3.00, *SD* = 1.45). Finally, social media news media usage was assessed with the item, “social media platforms such as Facebook, Twitter, and YouTube” (*M* = 3.11, *SD* = 1.53).

Control variables. Demographic characteristics were also incorporated into the analysis, including age, gender, ethnicity, and education level. We also included additional variables that may be related to emotional distress during the pandemic, such as (a) the likelihood of getting infected with COVID-19 as measured on a 5-point scale from 1 = *very unlikely* to 5 = *very likely* (*M* = 2.60, *SD* = 1.11), (b) whether participants knew someone likely to suffer serious negative consequences if infected with COVID-19 (*yes* = 1275, 58.1%; *no* = 921, 41.9%), and (c) whether they knew someone who has tested positive for COVID-19 (*yes* = 326, 14.8%; *no* = 1870, 85.2%). In addition, a measure of political ideology, measured on a 5-point scale from 1 = *liberal* to 5 = *conservative* (*M* = 3.06, *SD* = 1.08), was included in the analysis. Table 1 presents descriptive statistics and Pearson correlation coefficients among the variables.

### 2.3. Analytic Strategy

Hierarchical linear regression analysis was performed to examine the proposed hypotheses. The analysis was conducted in four steps. Emotional distress was entered as a continuous dependent variable; control variables including demographics, likelihood of getting infected, whether participants knew someone likely to suffer serious negative consequences or who has tested positive for the COVID-19 coronavirus, and political ideology were entered in Step 1. Information seeking about COVID-19 was entered in Step 2. The three news media use variables for television, newspapers, and social media were entered in Step 3 (to address possible multicollinearity between our multiple news media use terms, we also tested versions of the same model where we added each news media use variable and each interaction term separately. We confirmed that the results held). Finally, the interactions between information seeking about COVID-19 and the news media use measures were entered in Step 4. All predictors were mean-centered before they were entered in the moderated regression model. The analysis was conducted using SPSS version 26 (SPSS Inc., Armonk, NY, USA).

## 3. Results

Among the control variables, age and gender were significant predictors of emotional distress. Younger (β = −0.145, *p* < 0.001) females (β = 0.130, *p* < 0.001) were more likely to be emotionally distressed. Higher levels of distress were reported when people perceived higher likelihood of getting infected by COVID-19 (β = 0.178, *p* < 0.001) and if they knew someone who was high risk (β = 0.054, *p* < 0.01). Moreover, people with conservative ideology were less likely to be distressed (β = −0.068, *p* < 0.01).

Regarding H1, results revealed that while accounting for a variety of control variables, the more COVID-19 information individuals sought the more likely they were to be emotionally distressed (β = 0.255, *p* < 0.001; see Table 2). Thus, H1 was supported.

For H2, H4, and H6, even after statistically controlling for several variables, including COVID-19 information seeking, consuming news via television and social media was related to increased emotional distress (β = 0.099, *p* < 0.001 and β = 0.137, *p* < 0.001, respectively), whereas consuming news via newspapers was not (β = 0.032, *p* = 0.132). Thus, H2 and H6 were supported, but H4 was not.

With respect to H3, H5, and H7, findings indicated that emotional distress was significantly higher among those high in COVID-19 information seeking and television news use (β = 0.046, *p* = 0.033). There was no significant interaction between information seeking about COVID-19 and either newspaper use or social media news use (β = −0.002, *p* = 0.917 and β = 0.017, *p* = 0.393, respectively). This result provides support for H3 but not H5 or H7.

**Figure 1:**
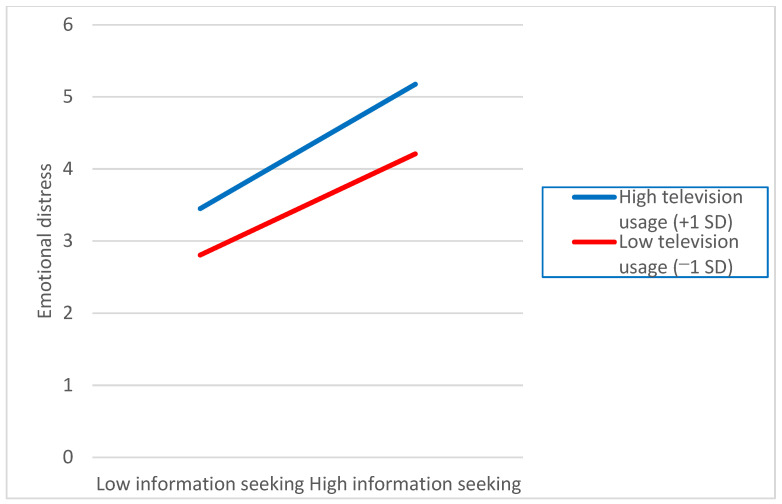
Interaction between information seeking and television news usage on emotional distress.

To understand the nature of this interaction, we plotted the interactive relationships between COVID-19 information seeking and television news use. These relationships are presented in Figure 1, which shows that the emotional distress experienced by those seeking COVID-19 information was further amplified among television news consumers. Thus, H3 was supported.

## 4. Discussion

The rapid emergence of COVID-19 has caused considerable psychological stress in the global population [2,6,7]. People seek information about the pandemic and follow the news to keep updated. We set out to understand the relationships among information seeking concerning COVID-19, general news media use, and emotional distress during the early stages of the pandemic, with a focus on media modality.

Our primary findings reveal that the more individuals sought COVID-19 information, the more likely they were to be emotionally distressed. Moreover, after accounting for COVID-19 information seeking, consuming news via television and social media was related to increased distress, while consuming newspapers was unrelated to distress. Our moderation analysis revealed that active COVID-19 information seekers who relied on television news were more likely to be emotionally distressed, but the association between COVID-19 information seeking and emotional distress was not amplified by newspaper or social media news use.

These findings contribute to the literature on several fronts. First and foremost, we advanced research on information seeking and emotional response by focusing on information seeking about a novel virus, which has resulted in an unprecedented global burden. The positive association between information seeking and emotional distress during the COVID-19 pandemic is reflective of this unique situation. It is notable that the positive association between information seeking and emotional distress remained significant when the three news sources were added to the model. There could be multiple possible reasons for these findings. First, while information seeking normally reduces uncertainty [55,56], COVID-19 information seeking likely increases uncertainty and anxiety because answers to basic questions, like when the pandemic will end, how the virus is transmitted, and its specific short-term and long-term impact remain unavailable. Although “ignorance may be bliss” from an emotional standpoint, the emotional distress concerning COVID-19 may be adaptive, possibly increasing protective health measures. In late March, the COVID-19 information available was quite limited, and centered on hand washing and social distancing recommendations, the lack of personal protective equipment and other medical equipment, and the increasing number of hospitalizations and deaths.

Next, our findings indicated that consuming news via television was related to increased emotional distress. Moreover, our moderation analysis revealed that people who sought COVID-19 information and viewed more television news tended to be even more emotionally distressed. Television’s vivid imagery and sound make it an emotionally arousing medium, so television news users may have a higher likelihood of experiencing distress when COVID-19 information seeking. These findings are consistent with previous research showing a strong association between television news and negative emotions during times of crisis, such as September 11 (e.g., [17]) and natural disasters (e.g., [57]). Our results suggest that the effect of television news on negative emotions can be applied to COVID-19.

In addition, our findings indicate that the more people consumed news from social media, the more likely they were to be emotionally distressed. This again could be due to the modality of social media, given it often combines text, audio, and video. The heightened distress among social media news users could also be due to misinformation and exaggeration of risks [53] and unverified contending opinions about an issue, which may heighten uncertainty [58,59,60]. The political nature of COVID-19 [61] means there is an immense amount of disagreement on social media platforms, extending to the very existence of the virus [62]. In addition, the fact that we found no interaction effects between information seeking and social media use on emotional distress could imply that the distress caused by social media may not be driven by information seeking but by other types of social media uses such as social interactions.

Finally, while we expected that consuming news via newspapers would be related to lower distress, given the less emotionally arousing modality and lesser partisan reporting style, our results revealed no significant association between newspapers and distress. This result could reflect that news users’ heightened stress during this pandemic was not accentuated by print media. Taken together, these results suggest that people who relied on television—and to a lesser extent social media—for news were more likely to experience emotional distress concerning COVID-19.

To sum, our findings show that people should be careful about their information gathering habits. We would recommend moderating media exposure because repeated media usage, especially via television news [22,43,44,45,46] may lead to heightened stress. Individuals should also take caution while gathering pandemic news from social media. Of course, the pandemic necessitates that we stay updated with the news for our own safety and the safety of those around us, but thoughtful information gathering and news consumption habits will perhaps facilitate better emotional health.

### Limitations and Future Directions

As with all research, our study comes with caveats. Due to the cross-sectional nature of the study, we cannot draw conclusions concerning causal relationships. It is also possible those with more emotional distress are more likely to seek COVID-19 information. Moreover, although we attribute the positive association between information seeking and emotional distress to unique features of COVID-19 information, such as persistent uncertainty, ubiquitous news coverage, and topic unavoidability, it is possible that information seeking could cause higher emotional distress only immediately; in the long-term, the emotional distress could become weak, possibly because people might gain a sense of control. However, prior research shows that in times of crises, information seeking can lead to emotional distress (e.g., [17,21,22,43,44,45,46]). Our findings support this phenomenon. Despite our justification, future studies should use longitudinal data to confirm causal relationships.

Related to this, it would be important to statistically control for media use level before the pandemic, since some people might increase their media use at the onset of the pandemic with others’ media use remaining static. Similarly, it would be ideal to measure the extent to which emotional distress was changed due to the emergence of the pandemic. Due to the lack of those pre-COVID measures in our dataset, however, we were not able to add those control variables in our model. Future studies should measure pre-pandemic values for primary behavioral variables to understand the dynamics of behaviors caused by the pandemic.

Additionally, our measurement of emotional distress only tracked those feeling overwhelmed, anxious, and afraid about what might happen. Given that emotional distress can also be linked to feeling depressed, worried, and sad, future studies should encompass more specific emotions with valid measurement. Moreover, we measured COVID-19 information seeking with a single item. Although our item clearly captured the extent of information seeking with regard to COVID-19, future studies should check the validity of the variable using a multi-measure approach that attends to exposure and attention in additional to information seeking. Similarly, while newspapers and news magazines may feature different characteristics, we measured them within an item, not differentiating those two. Also, although television news includes a variety of cable channels, including highly partisan outlets, we measured television news with national news networks. Future studies should define television news more broadly with more robust measurement.

## 5. Conclusions

Since the pandemic began, COVID-19 has dominated the news cycle [27,63]. Moreover, along with the pandemic, there has been another attack on the public, termed the “infodemic” [64] as people have been exposed to an abundance of false information. People are maneuvering this media environment to get information and manage the emotional stress they are feeling. Our study takes a preliminary step toward examining the association between information seeking, use of various types of news media, and emotional health during the early days of the COVID-19 pandemic. Examining emotional health is crucial in this situation, when people were primarily inside their homes and away from friends and family for months on end. The toll of this pandemic will not only be measured in terms of the loss of life, the long-term medical consequences, or the economic impact, but in terms of the emotional toll on the public.

## Figures and Tables

**Table 1:** Descriptive statistics and correlations.

Variables	*M*	*SD*	1	2	3	4	5
1. Emotional distress	3.45	1.08	—				
2. Information seeking about COVID-19	3.77	1.12	0.361 ***	—			
3. Television news	3.62	1.38	0.246 ***	0.359 ***	—		
4. Newspapers	3.00	1.45	0.177 ***	0.292 ***	0.370 ***	—	
5. Social media news	3.11	1.53	0.311 ***	0.210 ***	0.141 ***	0.143 ***	—

*Note. M* denotes mean; *SD* denotes standard deviation. *** *p* < 0.001.

**Table 2:** Hierarchical regression analysis examining the relationships between COVID-19 information seeking, news media usage, and emotional distress.

	Emotional Distress (β)
Block1: Control variables	
Age	−0.145 ***
Gender (Female = 1)	0.130 ***
Ethnicity (Minority = 1)	0.029
Education	−0.034
Likelihood of getting infected	0.178 ***
Know someone who is in high risk (yes = 1, no = 0)	0.054 **
Know someone who has tested positive (yes = 1, no = 0)	0.004
Political ideology (1 = liberal to 5 = conservative)	−0.068 **
Δ*R*^2^	9.2%
Block2: Information seeking	
COVID-19 Information seeking	0.255 ***
Δ*R*^2^	11.3%
Block3: News media usage	
Television news	0.099 ***
Newspapers	0.032
Social media	0.137 ***
Δ*R*^2^	3.4%
Block4: Interactions	
Information seeking × Television news	0.046 *
Information seeking × Newspapers	−0.002
Information seeking × Social media	0.017
Δ*R*^2^	0.4%
Total *R*^2^	24.3%

*Note.* All of the coefficients are standardized. Predictors (information seeking and news media usage) are mean-centered. ΔR^2^, the R square change, shows the improvement in R-square when the next group of predictors is added. * *p* < 0.05, ** *p* < 0.01, *** *p* < 0.001.

## Data Availability

The data presented in this study are available on request from the corresponding author.
